# Mid-upper arm circumference as a screening tool for identifying underweight adolescents

**DOI:** 10.3389/fnut.2023.1200077

**Published:** 2023-10-06

**Authors:** Imad R. Musa, Saeed M. Omar, Ashwaq AlEed, Abdullah Al-Nafeesah, Ishag Adam

**Affiliations:** ^1^Royal Commission Hospital at AL Jubail Industrial City, Al Jubail, Saudi Arabia; ^2^Faculty of Medicine, Gadarif University, Gadarif, Sudan; ^3^Department of Pediatrics, Unaizah College of Medicine and Medical Sciences, Qassim University, Unaizah, Saudi Arabia; ^4^Department of Pediatrics, College of Medicine, Qassim University, Buraydah, Saudi Arabia; ^5^Department of Obstetrics and Gynecology, Unaizah College of Medicine and Medical Sciences, Qassim University, Unaizah, Saudi Arabia

**Keywords:** body mass index, Z-score, mid-upper arm circumference, underweight, adolescents

## Abstract

**Background:**

Mid-upper arm circumference (MUAC) is a potentially credible alternative method for body mass index (BMI) to assess nutritional status. We aimed to assess the correlation between MUAC and BMI- Z-score and to identify a reliable MUAC cut-off point to detect underweight (BMI- Z-score of < −2 standard deviation) Sudanese adolescents.

**Methods:**

A cross-sectional study was conducted in eastern Sudan. After obtaining adolescents’ age and sex, their weight, height, and MUAC were measured using the standard procedures. The MUAC (cm) cut-off corresponding to underweight was calculated using receiver operating characteristic (ROC) curve analysis.

**Results:**

In total, 390 adolescents were enrolled in the study and 205 (52.6%) of them were females. The median (interquartile range, IQR) age was 15.1 (14.0–16.3) years. The medians (IQR) of MUAC and BMI- Z-score were 22.0 (20.0–24.0) cm and − 0.62 (−1.5–0.3), respectively. MUAC was positively correlated with BMI Z-score in all participants (*r* = 0.534, *p* < 0.001), in females (*r* = 0.715, *p* < 0.001), and in males (*r* = 0.404, *p* < 0.001). Of the 390 enrolled adolescents, 61(15.6%) were underweight. The MUAC cut-off for underweight was ≤21.2 cm in all participants (Youden’s Index, YI = 0.50; sensitivity = 82.0%; specificity = 68.0%, AUROCC = 0.78), in females (YI = 0.66, sensitivity = 86.0%, specificity = 80.0%, AUROCC = 0.87), and in males (YI = 0.32, sensitivity = 80.0%, specificity = 52.0%, AUROCC = 0.69).

**Conclusion:**

MUAC has good accuracy results and can be adopted for community-based screening of underweight adolescents.

## Introduction

Adolescence is defined by “The World Health Organization (WHO)” as an age between 10 and 19 years. Adolescents constitute 16% of the global population and the majority (90%) live in low-and middle-income countries (1, 2). Adolescence represents the period of development that starts at puberty and ends at adulthood, which reflects the physiological pattern (3). Adolescents are vulnerable to several risk factors for adult non-communicable diseases, communicable diseases, nutritional diseases, and malnutrition (4). Malnutrition among adolescents is associated with several medical problems such as increased risk of contracting communicable diseases, delayed growth, lower intellectual quotient, impaired cognitive maturation, and behavioral problems (5). Undernutrition is associated with poverty, violence, food insecurity, impaired sexual and reproductive health, and risk of contracting communicable and non-communicable diseases (3). Recent data showed that children and adolescents were at risk of malnutrition globally, and it was among the main causes of mortality: 225,906 deaths in 2013 (approximately 34 deaths per 100,000) which significantly varied between developing and developed countries with 38.5 per 100,000 and 0.2 per 100,000, respectively, (2).

Several anthropometric measurements such as weight, height, weight for height, head circumference, body mass index (BMI), mid-upper arm circumference (MUAC), and triceps skinfold thickness have been proposed to assess nutritional status (6). BMI may be affected by fluid overload, edemas, and muscle mass (7). However, the MAUC is a practical and cheap method. It is a simple measurement as it does not require difficult tools or expert frontline professionals compared to the requirements for measuring BMI (8). MAUC may be useful instead of BMI in different medical problems such as cancer, growth failure (9), among pregnant adolescents (10), and cerebral palsy (11). MUAC could be used as an alternative to BMI to evaluate nutritional status among adolescents in different countries, especially in countries with low resources (8, 12–14).

Malnutrition is a significant health problem in Sudan and it has economic, educational, and productivity impacts (15). There are no published data on the reliability of MUAC measurement in detecting the nutritional status of Sudanese adolescents. Thus, obtaining specific MUAC cut-offs for certain populations of adolescents could be an important method in countries with fewer resources like Sudan. Moreover, Sudan has suffered and is still suffering from civil war, tribal tension, famines, displaced people, and refugee crises, and thus requires a practical, simple, and cheap method to assess nutrition status. We aimed to assess the correlation between MUAC and BMI- *Z*-score and to identify a reliable MUAC cut-off point to detect underweight (BMI- *Z*-score of < −2 standard deviation) Sudanese adolescents.

## Methods

The Strengthening the Reporting of Observational Studies in Epidemiology (STROBE) standard checklists were followed (16). The methods followed in this study have been previously described in our previous work among adults with the same objectives. In summary: a multistage sampling study was conducted in eastern Sudan (New Halfa) during the period of January to February 2021. Four out of seven sectors, which are the lowest administrative units, were selected using a simple random method. The total sample size of 390 participants (both males and females) was distributed between the selected four sectors according to the size of the sector itself. Then, adolescents (10–19 years of age) and their parents in households were selected via a lottery method. If there was no adolescent in the selected household or they refused to participate or had met one of the exclusion criteria, the next household was chosen.

### Inclusion criteria

Apparently healthy adolescent males and females who were residents in the area of the study, whose age was (≥10 years – ≤19 years), and who had signed consent by their guardians for participation.

### Exclusion criteria

Age below 10 years and above 19 years; adolescent pregnant women; adolescents with chronic diseases such as diabetes, thyroid diseases, and heart failure; critically ill patients with severe acute illness; athletes; those on hormonal medication; those who had any apparent congenital dysmorphism; adolescents on chronic medications; and those who refused to participate.

The eligible adolescents were interviewed and their sex and birth dates (confirmed from identity cards) were recorded. Anthropometric measures (weight, height, and MUAC) were taken twice and the mean of measurements was taken. The measurements followed the standard procedures using calibrated instruments. A third measurement was performed in case of considerable variation between the first two readings (differences of >100.0 g for weight, 0.5 cm for height, and 0.2 cm for MUAC). After taking off their shoes and removing heavy clothing and objects from their pockets, the participants were then weighed (to the nearest 10.0 g). Their standing height was measured (to the nearest 1 mm) by a stadiometer with their feet positioned together at the heels with the back of the heels. MUAC was measured (to the nearest 1 mm) in sitting or standing posture using a non-stretchable MUAC measuring tape which was placed at mid between the olecranon process of the left ulna and the acromion process of the left scapula. BMI was computed as weight in kg/height in m^2^ (17). Thereafter, BMI Z-scores were calculated using the WHO international growth reference data for children and adolescents (18).

### Sample size calculation

A sample of 390 adolescents was calculated to obtain the significant minimum difference in the correlation (*r* = 0.15) between BMI Z-scores and MUAC. The sample (390 adolescents) had an 80% power and a difference of 5% at *α* = 0.05 (19).

### Statistical analysis

Data were analyzed using IBM SPSS version 25. Shapiro–Wilk tests were used to check the normality of the continuous data. A non-parametric Mann–Whitney *U* test was used to assess the difference in variables between the groups (males and females). Scatterplots with fitted linear regression lines were computed to evaluate the association between MUAC and BMI z-score for all adolescents and for each sex separately. The sensitivity and the specificity were computed and Youden’s Index (YI) was calculated as YI = sensitivity + specificity-1. The MUAC cut-off with the highest YI-value represented the optimal statistically-derived cut-off (20). The area under the receiver operating characteristic curve (AUROCC) was obtained for all adolescents and for females and males separately. A value of *p* less than 0.05 was considered statistically significant.

## Results

In total, 390 adolescents were enrolled in the study and 205 (52.6%) of them were females. The median (IQR) age was 15.1 (14.0–16.3) years. The medians (IQR) of MUAC and BMI- Z-score were 22.0 (20.0–24.0) cm and − 0.62 (−1.5–0.3), respectively. Compared with males, females were significantly older, taller, and had higher MAUC and BMI Z-scores (Table 1).

**Table 1:** Comparing medians (interquartile range) of anthropometric profile between adolescent boys and girls in eastern Sudan, 2021.

	Total (number = 338)	Female (number = 205)	Male (number = 184)	*p*
Age, years	15.1 (14.0–16.3)	15.6 (14.4–16.5)	14.5 (13.7–15.6)	0.031
Body mass index, Z score	−0.62 (−1.5–0.3)	−0.5 (−1.3–0.0.2)	−0.8 (−1.8–0.6)	0.617
Mid-upper arm circumference, cm	22.0 (20.0–24.0)	23.0 (21.0–25.0)	21.0 (19.0–23.0)	< 0.001

**Figure 1:**
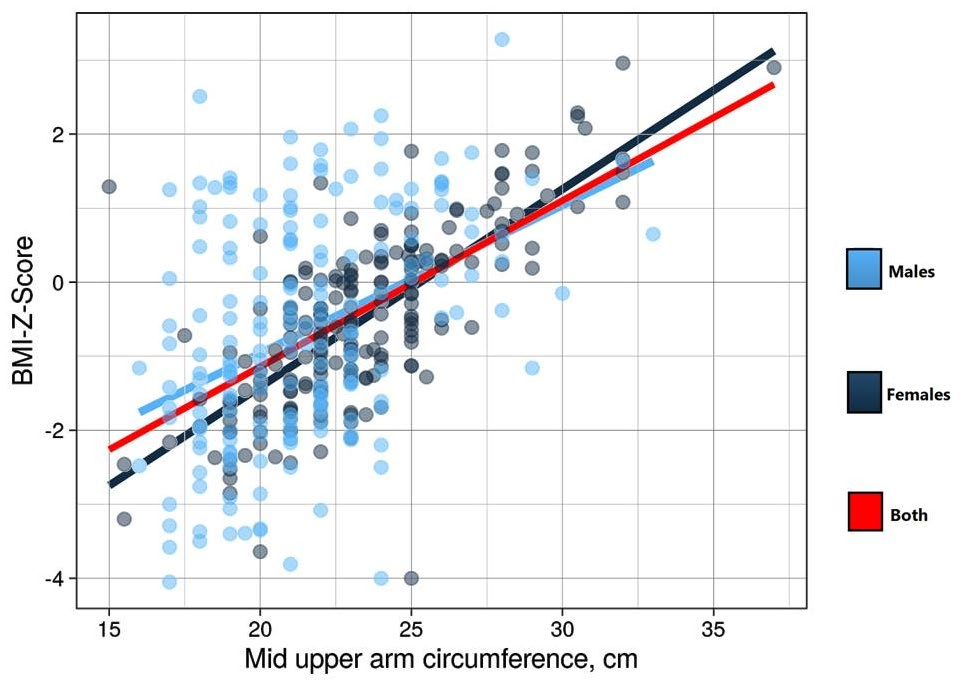
Curve estimation for assessing the linear relationship between mid-upper arm circumference and body mass index Z-score in adolescents in eastern Sudan 2021.

There was a significant positive correlation between MUAC and BMI Z-score (*r* = 0.534 (*p* < 0.001) in all participants, in females [*r* = 0.715 (*p* < 0.001)], and in males [*r* = 0.404 (*p* < 0.001; Figure 1)].

**Figure 2:**
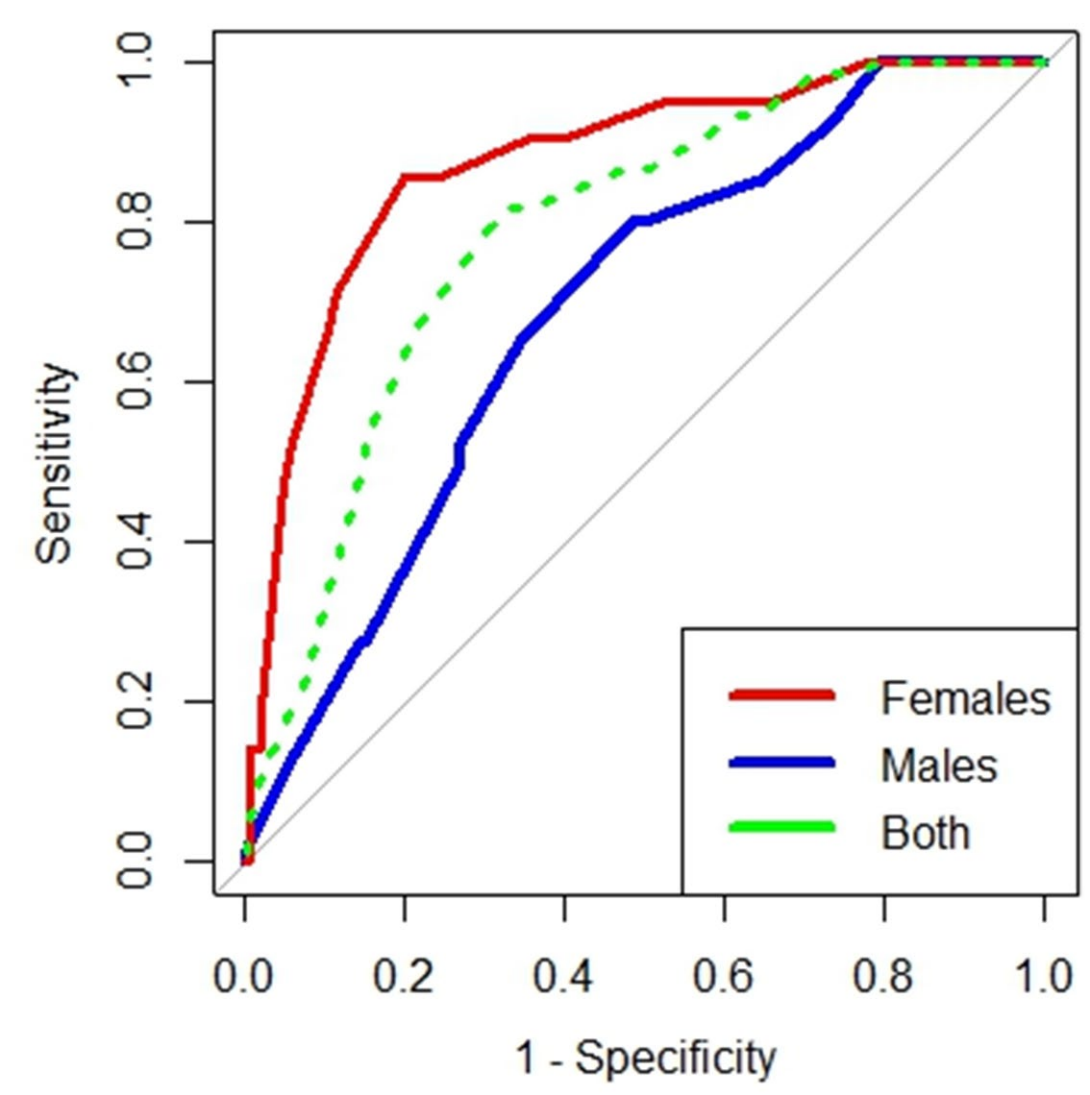
Receiver operating curve of mid-upper arm circumference for diagnosing underweight adolescents in eastern Sudan 2021.

Of the 390 enrolled participants, 61(15.6%) were underweight. The best statistically derived MUAC cut-off based on BMI z-scores for underweight was ≤21.25 cm in all participants (YI = 0.50; sensitivity = 82.0%; specificity = 68.0%) with a good predictive value (AUROCC = 0.78, 95.0% CI = 0.73–0.84), in females (YI = 0.66, sensitivity = 86.0%, specificity = 80.0%), (AUROCC = 0.87, 95.0% CI = 0.79–0.95), and in males (YI = 0.32, sensitivity = 80.0%, specificity = 52.0%), with a good predictive value (AUROCC = 0.69, 95.0% CI = 0.60–0.77; Table 2; Figure 2).

**Table 2:** Mid-upper arm circumference cut-off points for the diagnosis of underweight adolescents in eastern Sudan, 2021.

Variables	All participants	Females	Males
Mid-upper arm circumference cut-off	≤21.2 cm	≤21.2 cm	≤21.2 cm
Area under the curve (95.0% confidence interval)	0.78 (0.73–0.84)	0.87 (0.79–0.95)	0.69 (0.77–0.88)
Youden’s index	0.50	0.66	0.32
Sensitivity	82.0%	86.0%	80%
Specificity	68.0%	80.0%	52.0%

## Discussion

Our study showed a positive correlation between BMI Z-scores and MUAC in all adolescents and in each sex separately. This goes with similar previous findings reported among adolescents in Ethiopia (*r* = 0.81) (13) and in Tanzania [females (*R* = 0.846) versus males (*r* = 0.459)] (21).

The best statistically derived MUAC cut-off for underweight adolescents in this study was ≤21.2 cm which showed similar results in females and males separately. The MUAC cut-off observed in our results to detect underweight adolescents was almost similar to that obtained in Tanzania in adolescents (18.5–22.0 cm, sensitivity = 40.0%, specificity = 92.5%) aged 15–17 years old (21) and in India (≤21.6 cm, sensitivity = 75.4%, specificity = 87.1%, and AUROCC = 0.91; age 15–19 years) (12). In Tanzania (21), they enrolled adolescents aged 15–17 years old and we enrolled adolescents aged ≥10 years –≤19 years and this could explain the difference in our results and the results from Tanzania. However, the MUAC cut-off which is proposed to detect underweight in the current study is slighter higher than that reported in Turkey for males (≤20.50 cm sensitivity = 60.9%, specificity = 87.4%, and AUROCC = 0.791) and females (≤20.50 cm sensitivity = 45.0.8%, specificity = 87.8%, and AUROCC = 0.748) (22), in India (≤19.4 cm sensitivity = 84.0%, specificity = 81.4% and AUROCC = 0.86) for females aged 10–14 years (12) and in another study for males (≤19.2 cm sensitivity = 82.2%, specificity = 68.9% and AUROCC = 0.77) and females (≤19.4 cm sensitivity = 87.7%, specificity = 70.9% and AUROCC =0.79) aged 10–14 years (14). Additionally, a markedly higher MUAC cut-off of more than 21.25 cm was reported among adolescents in Ethiopia (≤23.3 cm, sensitivity = 87.9%, specificity = 75.9% and AUROCC = 0.90) for males and (≤22.6 cm, sensitivity = 100% specificity = 88.2% and AUROCC = 0.97) for females (13), in India (≤22.0 cm sensitivity = 77.0%, specificity = 79.6% and AUROCC =) in adolescents aged 15–19 years (8), and in males (≤22.9 cm sensitivity = 81.9%, specificity = 75.1% and AUROCC = 0.82) and females (≤21.7 cm sensitivity = 87.9%, specificity = 77.8% and AUROCC = 0.84) aged 10–14 years (14). The different MUAC cut-off points for different populations are summarized in Table 3. The variation in MUAC cut-offs in different studies may be explained by the difference in adipose tissue as well as in skeletal muscle mass observed in some children which could be due to differences in ethnicity (23). Moreover, a significant difference according to sex and age in undernutrition was observed in some studies (24–26). Hence, no universal agreement on the MUAC cut-off point to screen malnutrition among children and adolescents (27, 28). Therefore, adopting local references of MUAC for epidemiological and anthropological studies is recommended (29). Several previous studies reported that MUAC could be an alternative tool to detect underweight/thinness among adolescents (8, 13, 21, 27–29). MAUC is useful in different clinical situations, e.g., in cases of adolescents who have edema (7), chronic disorders and growth failure (9, 30, 31), cerebral palsy (11) severe learning disabilities (32), and anorexia nervosa (33, 34). Additionally, MUAC has also been adopted for many years for assessing nutritional status in certain conditions, like famines or refugee crises, where ordinary methods for height and weight measurements are difficult to perform (35). Furthermore, adopting a restricted MUAC cut-off for discharging children from severe acute malnutrition treatment can predict and prevent relapses and hospital readmission (36). Likewise, it has been shown to predict the worsening of nutritional status in low-income countries with laboratory indicators for hemoglobin, ferritin, zinc, serum albumin, and plasma retinol concentrations as dependent variables (37). Moreover, MUAC has emerged as the measurement that was most preferred by participants because it is less distressing than routinely used measurement techniques for weight and skin fold (34). Interestingly a simplified method of MAUC-based weight estimation can be used for the administration of many drugs and fluid regimens in emergency medicine that are weight-dependent in patients and when no standard adult weight estimation tools exist or are difficult to perform (27, 38, 39).

**Table 3:** Specificity, correlation coefficients and the area under the receiver operating characteristic curve (AUROC) from different studies on underweight adolescents using mid-upper arm circumference and body mass index Z-scores.

Study years/country	Total	Male	Female	Age	MUAC cut-off, cm	Youden’s Index	Sensitivity	Specificity	AUROCC	*r*
(11) (Ethiopia)	851	456	395	15–19	M 23.3 F 22.6	M 0.64 F 0.88	M 87.9 F 100.0	M 75.9 F 88·2	M = 0.90 F = 0·97	0.81
(21) (Tanzania)	154	62	92	10–14 15–17	16.0–18.5 18.5–22.0		40.0	92.5		M = 0.459 F = 0.846
(8) (India)	106,208	14,893	91,315	15–19	≤22.00		6.6	99.1		M = 0.54 F = 0.55
(12) (India)	2,492		2,492	10–14	≤19.4	0·59	84·0	75·4	0·86	0·780
(12) (India)	2,136		2,136	15–19	≤21.6	0·68	81·4	87·1	0·91	0·780
(14) (India)	31,471	16,158	15,313	10–19	M 21.9 F 20.4	–	–	–	–	0.810
				10–14	M 19.2 F 19.4	M 0.51 F 0.59	M 82.2 F 87.7	M 68.9 F 70.9	M 0.77 F 0.79	
				15–19	M 22.9 F 21.7	M 0.57 F 0.66	M 81.9 F 87.9	M 75.1 F 77.8	M 0.82 F 0.84	
(22) (Turkey)	626	307	319	10–13	M 20.50 F 20.50		M 60.9 F 45.8	M 87.4 F 87.8	M 0.791 F 0.748	

M, males; F, female; MUAC, mid upper arm circumference; AUROCC, Area Under the Receiver Operating Characteristics Curve; YI, Youden’s Index; *r*, correlation coefficient.

Our study is a community-based study and enrolled both males and females and these are strengths of this study. One limitation of the present study is that the data for MUAC cut-off from this area might be different from other areas in Sudan. Moreover, height, weight, and MUAC are all measurements that are subjected to measurement error due to inter-observer differences in measurement or miscalculation error (40). A larger sample size might have strengthened the prevalence of the outcome and the analysis.

## Conclusion

Our study proposes the cut-offs based on MUAC (≤22.5 cm) as an alternative for BMI for community-based screening of underweight adolescents.

## Data availability statement

The original contributions presented in the study are included in the article/supplementary material, further inquiries can be directed to the corresponding author.

## Ethics statement

The studies involving humans were approved by this study complies with the Declaration of Helsinki. Ethics approval was obtained from the Ethics Committee of the Faculty of Medicine of Gadarif University, Sudan (Reference number #2021.03). Written informed consent was collected from each participant. The studies were conducted in accordance with the local legislation and institutional requirements. The participants provided their written informed consent to participate in this study.

## Author contributions

IM and SO conceived the study and supervised data collection. AA, AA-N, and IA supervised the work, guided the analysis, critically reviewed the manuscript, prepared the analysis plan, performed the data analysis, and wrote the first draft of the paper. All authors reviewed and approved the final manuscript.

## Abbreviations

AUROCC: Area Under the Receiver Operating Characteristics Curve
BAZ: BMI-for-age Z-score
BMI: Body mass index
CI: Confidence interval
cm: centimeter
IQR: Interquartile range
kg: kilogram
ROC: receiver operating characteristic curve
m: meter
MUAC: mid-upper arm circumference
YI: Youden’s Index
SPSS: Statistical Package for the Social Sciences
STROBE: The Strengthening the Reporting of Observational Studies in Epidemiology
WHO: The World Health Organization
